# Automatic Frequency Controller for Power Amplifiers Used in Bio-Implanted Applications: Issues and Challenges

**DOI:** 10.3390/s141223843

**Published:** 2014-12-11

**Authors:** Mahammad A. Hannan, Hussein A. Hussein, Saad Mutashar, Salina A. Samad, Aini Hussain

**Affiliations:** 1 Department of Electrical, Electronic & Systems Engineering, Faculty of Engineering and Built Environment, Universiti Kebangsaan Malaysia, 43600 UKM Bangi Selangor, Malaysia; E-Mails: husseinalsaedi86@siswa.ukm.edu.my (H.A.H.); saad_ra25@yahoo.com (S.M.); salina@eng.ukm.my (S.A.S.); aini@eng.ukm.my (A.H.); 2 Department of Electrical and Electronic Engineering, University of Technology-Iraq, Baghdad 35010, Iraq

**Keywords:** bio-implanted devices, bio-telemetry systems, power amplifiers, automatic controller, inductive coupling link

## Abstract

With the development of communication technologies, the use of wireless systems in biomedical implanted devices has become very useful. Bio-implantable devices are electronic devices which are used for treatment and monitoring brain implants, pacemakers, cochlear implants, retinal implants and so on. The inductive coupling link is used to transmit power and data between the primary and secondary sides of the biomedical implanted system, in which efficient power amplifier is very much needed to ensure the best data transmission rates and low power losses. However, the efficiency of the implanted devices depends on the circuit design, controller, load variation, changes of radio frequency coil's mutual displacement and coupling coefficients. This paper provides a comprehensive survey on various power amplifier classes and their characteristics, efficiency and controller techniques that have been used in bio-implants. The automatic frequency controller used in biomedical implants such as gate drive switching control, closed loop power control, voltage controlled oscillator, capacitor control and microcontroller frequency control have been explained. Most of these techniques keep the resonance frequency stable in transcutaneous power transfer between the external coil and the coil implanted inside the body. Detailed information including carrier frequency, power efficiency, coils displacement, power consumption, supplied voltage and CMOS chip for the controllers techniques are investigated and summarized in the provided tables. From the rigorous review, it is observed that the existing automatic frequency controller technologies are more or less can capable of performing well in the implant devices; however, the systems are still not up to the mark. Accordingly, current challenges and problems of the typical automatic frequency controller techniques for power amplifiers are illustrated, with a brief suggestions and discussion section concerning the progress of implanted device research in the future. This review will hopefully lead to increasing efforts towards the development of low powered, highly efficient, high data rate and reliable automatic frequency controllers for implanted devices.

## 1. Introduction

Recently there have important technological developments in biomedical microsystems that have helped guide scientists to design small size, high efficiency and fading power consumption biomedical devices that can be implanted inside the human body using a surgical operation. Medical implanted devices sense directives of nerve signals from inside the human body in real-time, providing early diagnosis and treatment of diseases. Implanted medical devices used for diagnosis and treatment include retinal implants for the blind, pacemakers, cochlear implants for deafness, spinal cord stimulators for control of pain, deep-brain stimulators for Parkinson's disease, and brain-machine interfaces. Generally they keep the sufferer safe from the hazards of infection and improve his/her mobility.

Previously these devices were powered using wires that penetrate living tissue, and this may cause hazards and skin infections. In order to avoid these problems, researchers have developed implanted devices to be powered using batteries. The limited time-life, large size and side effects of this power source have led to new tactics to monitor and power the implanted devices such as thermal method, vibration method and coupling link method. Efficient and medically safe power sources are required to operate transcutaneous transfer systems. The best achievement in powering implantable devices involves the use of inductive coupling. Many applications have been developed for this wireless charging method, which differ in the chip fabrication method, operating frequency used and the transceiver coils.

**Figure 1.**
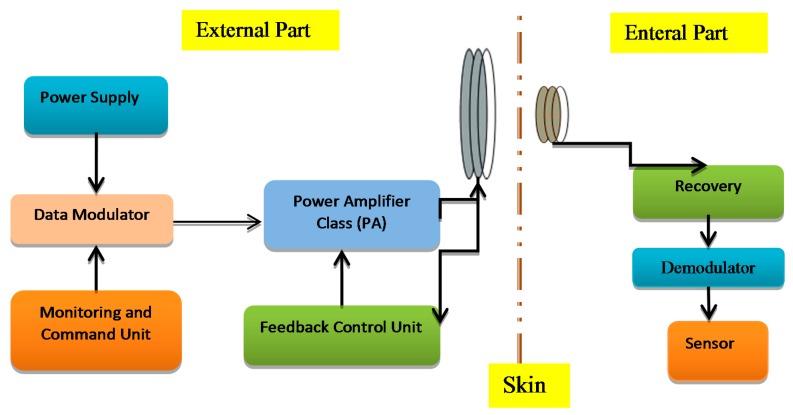
The biomedical transcutaneous system.

The transcutaneous transfer system has two parts: first is the external or primary part and the second part is the internal or secondary part implanted in the body tissue as shown in Figure 1. The external power unit produces a high frequency magnetic field in an external coil, through which the power is inductively conveyed to an internal coil that is associated with the implantable device. The modulated carrier is passed to the power amplifier to boost the signal power up to the level required for reliable communication [1]. The power amplifier ought to be highly efficient to minimize the power dissipation and lower power consumption. Implantable devices are supposed to work depending on the patient's condition so they do not use external sources of power. Therefore, a good circuitry design is required to satisfy biomedical implant device requirements like compact design, higher output gain, higher bandwidth, low cost and safety [2].

Radio frequency (RF) signals are used to operate the implanted devices inductively by coupling links. This makes the systems more reliable, flexible, more efficient and safer [3]. The adjustment of the operating frequency is very important in an inductive coupling system to prevent power and data loss so researchers have designed and simulated many techniques to control the resonance frequency to improve the efficiency in biomedical implant transmission systems. Many works related to the wireless low power frequency control electronics are presented such as transcutaneous power transfer systems [4], controlled coil drivers for transcutaneous power transmission [5], voltage controlled oscillators (VCOs) tuned to control an adaptive neural network [6], medical implants using hybrid coils [7] and wireless power regulation [8]. This paper provides a detailed review of the literature concerning the methods to regulate the power transferred over a wireless link by adjusting the resonant operating frequency of the external part of the system. These frequencies should use the Industrial Standard Medical (ISM) and Medical Implants Communication Systems (MICs) bands, which are employed for medical applications. The main reason for this review is to explain and clarify the methods used in frequency control of biomedical implanted devices to provide a good knowledge about the challenges and issues that are still exist and to find the best solutions.

## 2. Power Amplifiers for Transcutaneous Applications

The supply of wireless power to biomedical implants begins with power amplifiers, which supply energy at a particular frequency to an antenna or coil. As the size of implants decreases, so does the available space for receiving antennas. This leads to shorter wavelength antennas and lower size constraints for coils, which increases the frequency of power transmission. High transmission frequencies place a particular focus on utilizing power amplifiers, which operate efficiently at these levels. Switched power amplifiers have been a popular choice to drive inductive power links for implantable electronics due to their ability to minimize losses at high frequencies [9]. The power amplifier increases the incoming signal to the desired power level that drives the transmitting circuit.

### 2.1. Characteristic of Transcutaneous Power Amplifiers

The most used methods for energizing the transmitter coil in the external part of the biomedical implanted system is the use of a power amplifier. The power amplifier should satisfy the following three characteristics:

i. The active device drops a minimal voltage (it is supposed to be zero) while current is flowing through it.
ii. The active device is switched on when the voltage through it becomes zero, and switched off when the current through it is zero.
iii. The active device must switch fast enough to minimize the time when there is the simultaneous existence of current through the device and a significant voltage across it.

**Figure 2.**
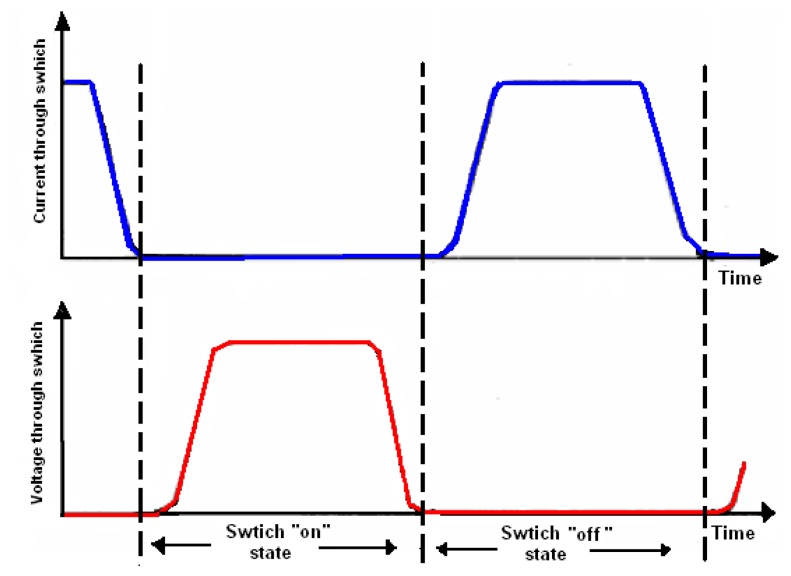
Transcutaneous power amplifier characteristics.

These characteristics are strongly focused on minimizing switching losses [10]. Figure 2 shows the switching activities of the transcutaneous power amplifier.

### 2.2. Amplifier Classes Used by Implanted Devices

**Figure 3.**
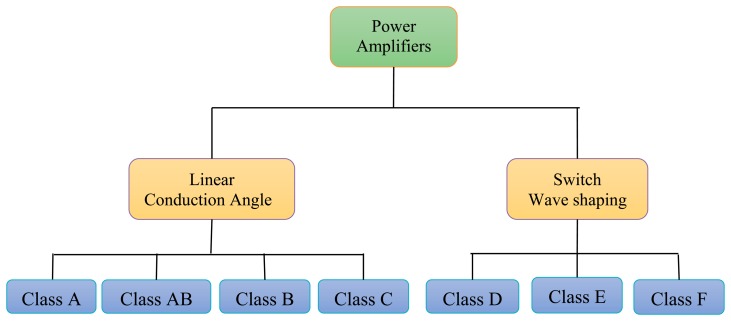
Power amplifier classes.

In transcutaneous power amplification systems different classes of power amplifiers (PAs) such A, AB, B, C, D, E, and F are used for the biomedical frequency bands. Output power ranges from a few mW to several Watts, depending on the applications. The bias applied to a power transistor determines the type of amplifier class [11]. The level of performance of a PA can be evaluated by choosing its bias point. From the PA bias, efficiency, linearity, or other parameters can be evaluated. The power amplifiers are divided into two main types, according to the operating mode [12] as shown in Figure 3. The first type is conduction angle or linear PA, if the PA transistor works as a converter of the RF input signal into a current. The second type is a switching PA, if the PA operates as a switch.

#### 2.2.1. Linear-Mode Power Amplifiers

The conduction angle gives the proportion of an AC cycle, which the output devices conduct [13]. The quiescent DC operating point (Q-point) of an amplifier defines the amplifier class. If the position of the Q-point at half of the load line of the amplifier's characteristic curve, the amplifier will operate as a Class-A amplifier with 360° conduction angle. Moving the Q-point lower down the load line changes the amplifier into a Class AB, B or C amplifier. A 180° conduction angle defines Class B, increasing it to 360° defines Class AB and when it is less than 180° it defines a Class C one.

**Figure 4.**
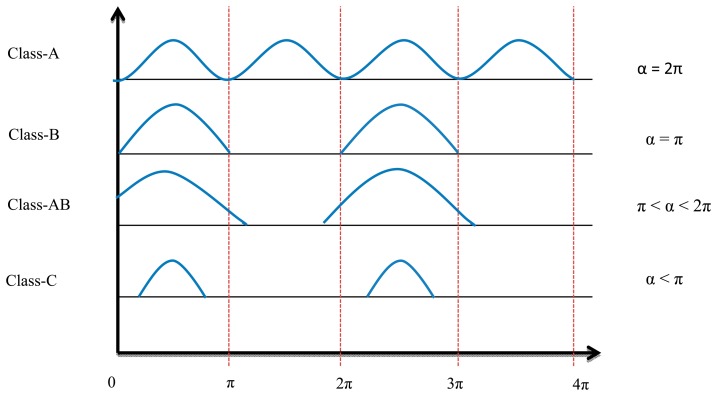
Conduction angle and input current signal for linearly-PA.

Figure 4 shows the conduction angle (α) and input current signal. Because of the low efficiency (compared with switch mode power amplifiers), high power losses, and low frequency band of linear power amplifiers, these elements are no longer used for transcutaneous power transfer systems.

The formulas below are used to calculate the conduction angle [14]:

(1) $$I_{d}=\;\{\begin{array}{ll} I_{q}+A_{\text{out}}.\cos\theta & \text{for}\;\theta \leq \alpha \;\text{and}\;\theta \;\geq 2\pi -\alpha \\ 0 & \text{for}\;\theta \;\text{otherwise}. \end{array}$$

(2) $$\cos\alpha =\;-\frac{I_{q}}{A_{\text{out}}}$$

(3) $$A_{\text{out}}=k\;A_{in}$$

where *I_d_*, is the current at the output of the transistor, *A_out_* is the magnitude of the sinusoidal peak current corresponding to a sinusoidal peak voltage excitation of magnitude *A_in_*, under a linear gain of *k*. Applying Fourier Series analysis to Equation (1), the following formulas for the current at the fundamental and DC are obtained:

(4) $$i_{RF}=\;\frac{1}{\pi }\int_{0}^{2\pi }i_{DC}\;\cos\left(\theta \right)\;d\theta =\;\frac{2}{\pi }\left(Iq\;\sin\alpha +\frac{A_{\text{out}}}{4}\sin2\alpha +\frac{\alpha \;.\;A_{\text{out}}}{2}\right)$$

(5) $$I_{DC}=\;\frac{1}{2\pi }\int_{0}^{2\pi }i_{DC}\;d\theta =\;\frac{1}{\pi }\left(Iq\;\alpha \;+\;A\text{out}\;\sin\alpha \right)$$

The maximum efficiency achieved for any given class of operation occurs when the voltage across the device is in voltage saturation. Then efficiency is determined by the ratio of the currents:

(6) $$\eta =\;\frac{i_{RF}}{I_{DC}}=\;\frac{\sin\;2\alpha -\;2\alpha }{4\;(\alpha \;\cos\alpha -\;\sin\alpha )}$$

#### 2.2.2. Switch-Mode Power Amplifiers

To operate the PA in switch-mode transistors are used to work in saturation mode. That means the voltage is equal to zero when the transistor is in the on state and the current equals zero when it is turned off, so there is no overlap time between voltage and current and for that reason they have the lowest power dissipation and achieve 100% theoretical efficiency. Therefore they are widely used in transcutaneous power amplification systems [15].

In practice a transistor is not a perfect switch and overlap does, in fact, limit efficiency. Switched power amplifiers use an output matching network (LC) that develops the waveform by isolating the harmonic components of the voltage and current. Only the fundamental current flows to the load and only the fundamental voltage is generated in the LC matching network. The two main conditions to produce signals with maximum efficiency in the load are as follows:

i. Zero crossing between voltage and current over the transistor channel.
ii. Isolate the fundamental harmonics of the current from pass to the load.

There are many kinds of switching-mode power amplifiers typified as Class F, Class D and Class E. The main differences between these classes are the topologies, waveform shaping, and method of analysis [16]. Class E power amplifiers are used widely in transcutaneous power systems because of their simplicity (they consist of only one transistor), high efficiency, wide frequency bandwidth and simple control circuitry [17].

##### Class F Power Amplifiers

**Figure 5.**
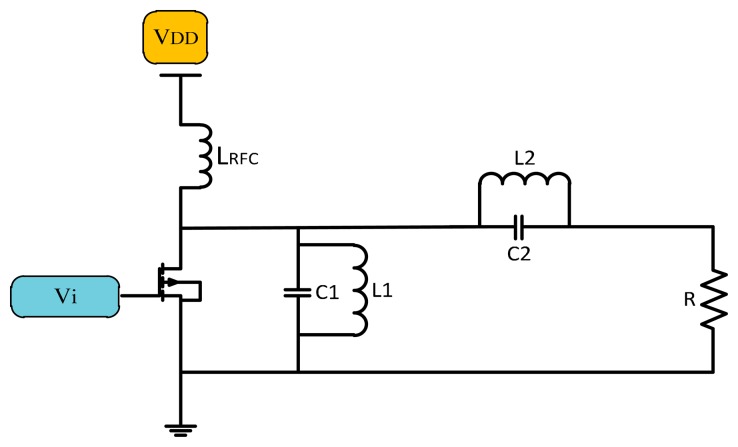
Class-F power amplifier.

The Class F power amplifier is a development of the Class E power amplifier that uses a harmonic resonance circuit on the load side [18]. A simple Class F amplifier design consisting of a transistor, choke inductor and an input source is shown in Figure 5. The resonance circuit on the output of the transistor cancels the harmonics generated by the system.

**Figure 6.**
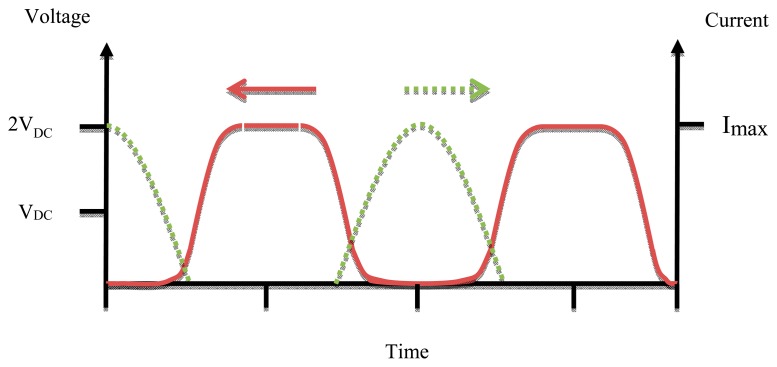
Class-F PA voltage and current waveforms.

The voltage and current waveforms of Class F amplifiers are shown in Figure 6, where the voltage is like a square wave due to the odd harmonics of the fundamental frequency. Current is like a half sine wave that has a phase degree of π [19]. Class F amplifiers are supposed to have a theoretical efficiency of 100%, but the resonant circuit become difficult to design for higher than 5th harmonics [20].

##### Class D Power Amplifiers

**Figure 7.**
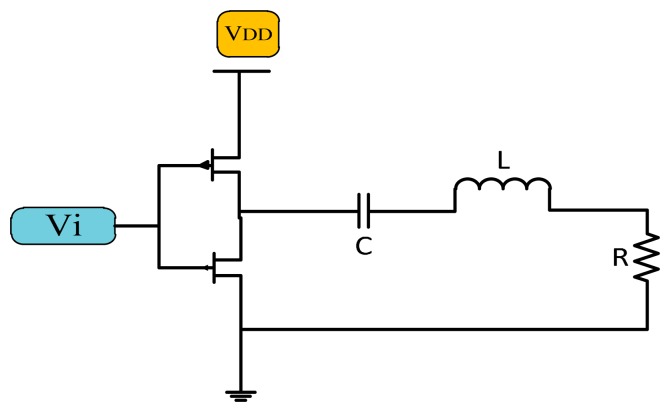
Class D power amplifier.

The Class D power amplifier switches two transistors on and off alternatively to generate a square wave. The output of the transistor is connected to the LC matching network as shown in Figure 7, which is resonant at the same resonance frequency of the square wave generating a sinusoidal signal of the same frequency [21].

**Figure 8.**
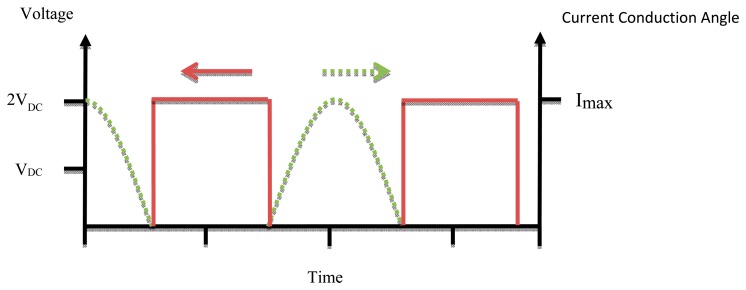
Class-D PA waveforms.

In the case that the matching network works properly the efficiency of the Class D amplifier ought to be 100%, but practically at high frequency the parasitic capacitance must be taken into account and the circuit component is not ideal so the efficiency is reduced. The size of Class D power amplifiers is bigger than that of Class F and E ones due to the use of two active devices [22]. Figure 8 shows the waveforms of voltage and current of Class D PAs.

##### Class E Power Amplifiers

**Figure 9.**
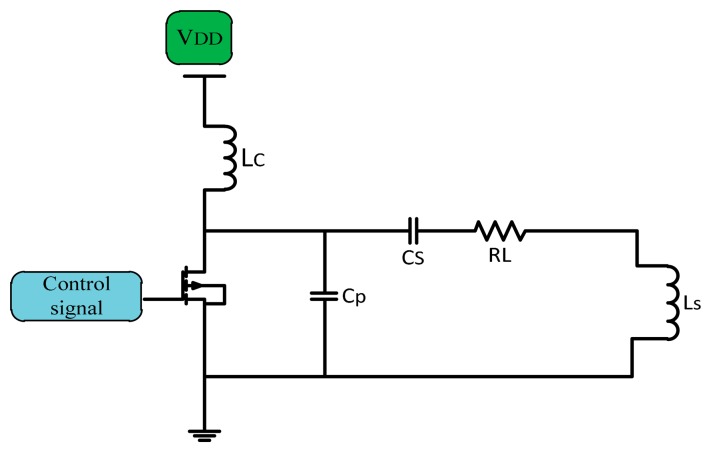
Class E power amplifier.

In implanted device transcutaneous systems Class E PAs are broadly used. They have a high efficiency near 90%, simple architecture, higher power transfer, no mixer is used if used as a modulator which make them better as power drivers for transmitter coils [23,24]. The simple Class E PA consists of a CMOS transistor as a switch, and a shunt capacitor (C) used for soft switching in non-ideal CMOS, so there is no overlap between voltage and current. A choke inductor (L_C_) used to keep supplied current (I_DC_) constant DC flows through the amplifier. The shunt capacitor C_P_ connected in parallel with the series load network of the transistor is adjusted to a known frequency to fix the current of the supply source and convert the digital input into a sinusoidal output with zero DC [25]. Figure 9 shows a simple Class E power amplifier.

**Figure 10.**
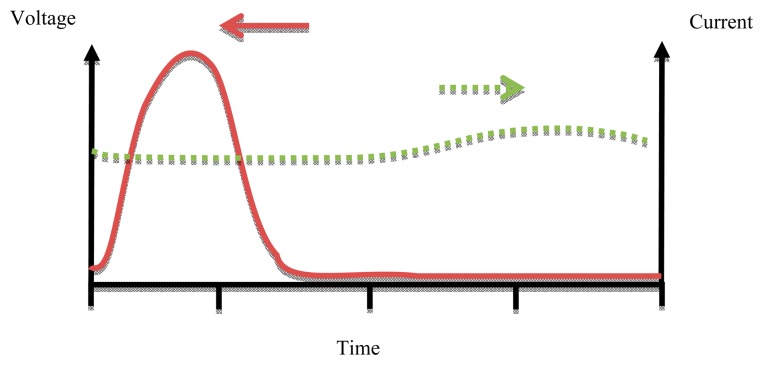
Class E waveforms.

A Class E power amplifier works at high operation frequency depending on the output capacitance required for the output matching circuit [26]. Maximum efficiency occurs at the high frequency limit of capacitance (Cs). Figure 10 shows the waveforms of voltage and current for a Class E PA.

Since the power amplifiers are used in biomedical implants many analyses and considerations have been made to develop the design of the Class E power amplifiers to make it more practical. One of these the Class E power amplifier designs was the infinitely loaded quality factor (Q). The Q of the output circuit LC must be high to ensure the output current and output voltages consist of only the fundamental harmonics [27]. The formulas below are used to design Class E power amplifiers [28]:

(7) $$P_{o}\;=\;\frac{2}{1+\frac{\pi^{2}}{4}}\;\cdot \;\frac{V_{CC}^{2}}{R}$$

(8) $$Q\;\leq \frac{\omega L}{R}$$

(9) $$C_{S}\;=\;\frac{1}{\omega_{o}R_{L}\;\left[\frac{\pi^{2}}{4}+1\right]\;\left[\frac{\pi }{2}\right]}=\;\frac{1}{\omega_{o}\left(5.447\;R_{L}\right)}$$

(10) $$C_{P}=C_{s}\;\left[\frac{5.447}{Q}\right]\;\left[1+\frac{1.42}{Q-2.08}\right]$$

### 2.3. Power Amplifier Efficiency

One of the most important performance parameters of an implantable wireless transmitter is its efficiency. It is a ratio of the power output of the PA over the input power of the PA represented as a percentage as shown in Equation (5) [29]:

(11) $$\text{Efficiency}\left(PA\right)=\frac{P_{o}\left(AC\right)}{P_{i}\left(DC\right)}\;\times 100\%$$

The major parts accounting for transmitter efficiency are power amplifier and antenna. A switching Class PA is more efficient than its linear class counterpart. It behaves as a switch such that current and voltage does not achieve their maximum value simultaneously which reduces power dissipation. Switching Class PAs requires the use of constant envelope modulation techniques such that the signal does not carry any amplitude information because of the aggressive amplitude compression experienced in switching PAs [30]. The Antenna Matching network accounts for a great part of the power losses. A good design of the matching network between PA and antenna is needed so that all the power will be delivered to the load. There is different architecture available for constructing a power amplifier that converts a DC voltage to AC voltage such as in A, B, AB, C, D, E, F and other PAs [31]. Efficiency does not represent a physical parameter; it's just useful for showing the requirements of the circuit for matching and stability. Table 1 explains the percentage efficiency, advantage and disadvantage for each class.

## 3. Automatic Frequency Controller

**Figure 11.**
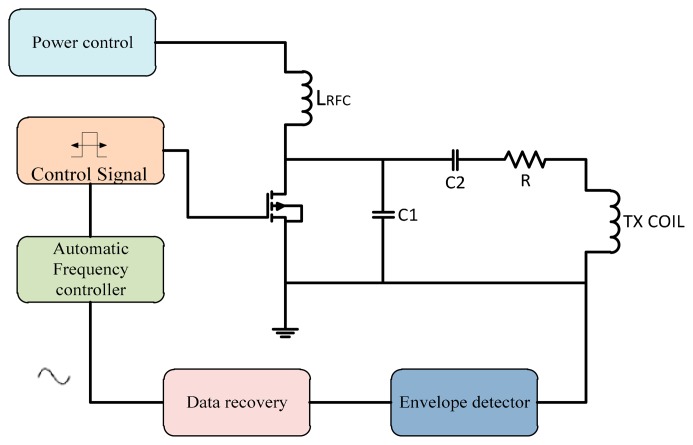
Automatic frequency control loop.

Wireless inductive coupling uses magnetic coupling as the communication environment that is common with radio frequency identification (RFID) techniques in the transcutaneous power system. Low power RF radiates RF power signals from the reader coil antenna, which is designed to send modulated signals stable in amplitude and frequency that are suitable to transmit power. The stability of the transmitted signal makes for a high readability for DC voltage at the implant device despite the mutual displacement between coils [32]. The bio-device system is composed of two coils: one implanted inside the human body, the other located outside the body. The best power transfer efficiency in transcutaneous systems occurs when the transmitter and receiver coils are tuned to the same resonant frequency *f*_0_. Misalignment or changes in the distance between the coils will cause a carrier frequency shift [33], so it is necessary for a frequency controller to correct the signal. The negative feedback-loop technique keeps the transcutaneous power transmission system at a stable voltage by regulating the supplied voltage, adjusting the duty ratio and correcting the frequency shift of the driving signal. The automatic frequency controller is shown in Figure 11.

### 3.1. Frequency Control Techniques Used in Biomedical Implants

In practice, there are various methods that can be implemented to monitor the RF transmitted signal in biotelemetry systems. The main important problem for PA efficiency is the resonant frequency control. The goal is to monitor and control the transmitted signal frequency of the power amplifier by using a frequency control technique. The advantage of this technique is offering a high transfer wireless power stability that maintains the stability of DC voltages at the implant device [34]. In this study, the methods which can be implemented for monitoring the RF transmitter power, using the frequency control techniques were investigated. Transcutaneous amplifiers commonly provide convenient power control abilities and are designed for particular applications but by controlling the resonant frequency between internal and external coils they will perform more efficiently, reliably and stably. These frequency control techniques require more circuit size, but because the issue deals with human systems stability is very important for human safety.

#### 3.1.1. Gate Drive Switching Control Closed Loop Technique

**Figure 12.**
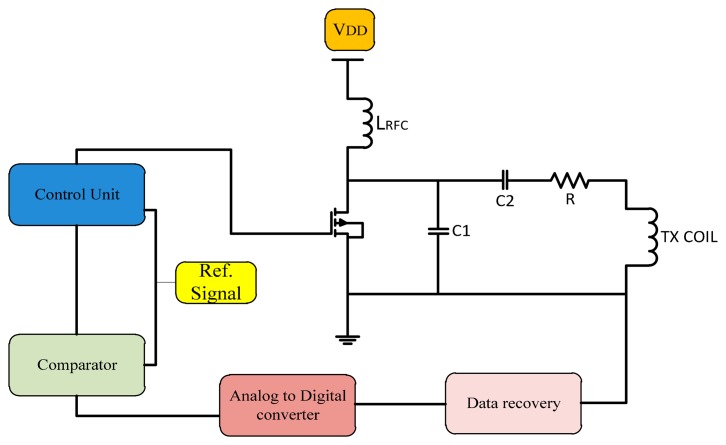
Gate drive switching control technique.

The closed-loop technique is used widely in transcutaneous systems for implantable devices to control the power amplifier frequency. In this method a feedback signal is applied to the gate of the power control transistor as shown in Figure 12. The proposed system is designed to provide the maximum power transfer to the implanted circuit. Circuits ought to tolerate coil misalignments and the changes in load current when the sensitivity to the device is minimized and accommodate process variation [35].

Gate drive switching as used by Troyk and Schwan [36] is a closed-loop control to correct the frequency in case of variations between transfers. Using coil current zero-crossing control provides excellent stability of the operating frequency even for changes in the coil inductance and resistance. Djemouai *et al.* [37] used a system that controls the output of the power amplifier (PA) by a feedback control circuit. The PA output stability is achieved by varying the current I_ctr_ between 4 μA and 6 μA. Baker and Sarpeshkar [38] used a feedback technique that reduced the use of algebraic manipulations. When transmission displacement varied from 1 to 10 mm the overall efficiencies were of 74% and 54%.

In 2009, Chen *et al.* [39] made a prototype transcutaneous power regulator that produces good load and input voltage regulation and high efficiency for variations of the alignment or gap in transcutaneous systems. Dissanayake *et al.* [40] designed a system with output power up to 15 W using a feedback frequency control that tolerate variations due to changes in alignment between transfer system coils. Yang *et al.* [41,42] used the feedback signal, compare it with a reference signal so the error signal healed and used to adjust the duty cycle and the angular frequency of the transmitted signal. Results showed that Class-E amplifier can work at maximum efficiency with feedback loops. Tang *et al.* [43] designed a transcutaneous power regulator for artificial hearts using modulation to control switching of duty ratio, so the efficiency was increased.

In 2010, Yang *et al.* [44] presented a feedback analysis of inductive links powered using a Class E amplifier with a variable coupling coefficient. Results showed that the inductive link tested in the best state with constant output voltage in the case where the duty cycle and the frequency of the driving signal were adjusted. A feedback controlled coil for transcutaneous applications was proposed to power the bio-implantable microsystems [45]. The ASK modulation technique is used to control the switch according to the input data by using an adaptive voltage doubler and rectifier [46]. To reduce the dropout voltage, Chan and Chen [47] adjusted high speed comparators that operate as switches at proper times in the high frequency band. Hence, the controlled Class E efficiency is varied between 30% and 66.2% when operating in the constant current mode. In 2013, Abu Khater *et al.* [48] used control methodology to smooth the transition between switching states based on pulse density modulation (PDM) feedback, as well as the output voltage slope-limiting circuit. The mechanism improves ripple reduction without any significant effect on efficiency, and with minimum off-chip components. Miura *et al.* [49] designed a transcutaneous energy transmission system for implantable internal artificial organ systems. The system was minimized and efficiency improved to 93.4% [50]. Valente *et al.* [50] designed Class D and Class Pas with a 0.18 μm CMOS.A closed-loop transmitter system to optimize the power delivery to medical implants so the efficiency was 80%. Summaries of frequency, power consumption, coils displacement and efficiency of gate drive switching frequency control are shown in Table 2.

#### 3.1.2. Power Control Closed Loop Technique

**Figure 13.**
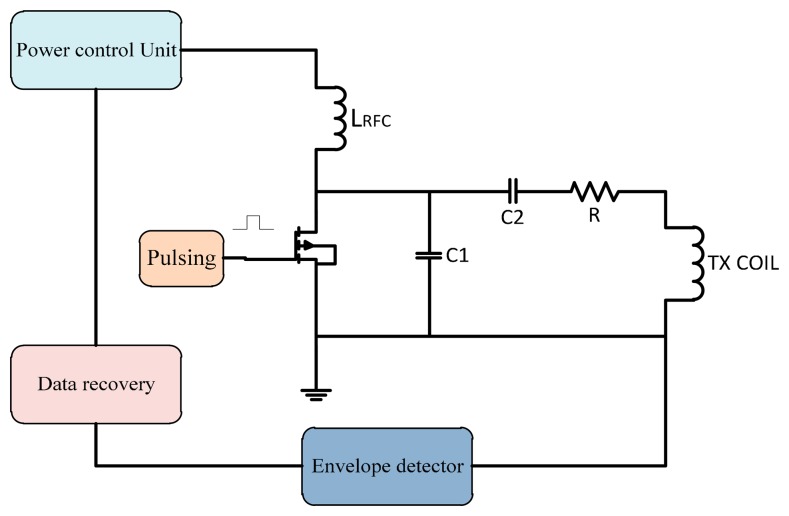
Power control technique.

Power control techniques can be used to improve the efficiency of the PA. In a switch mode PA, the input power should be stable so a supplied power control is required. In this technique the feedback signal back to the power control unit that controls the supplied power to the transcutaneous power amplifier to overcome the variation in frequency shift due to misalignment. This technique architecture is shown in Figure 13.

A power control unit was developed by Guoxing *et al.* [51] to ensure maximum power transfer to the implanted unit. MATLAB and Spice simulations were used in the tests. Simulations showed the validity of the system model. Guoxing *et al.* [52] designed a transfer system with a control technique that fixed the variations due to the load or coupling coefficient changes. The power supplied to implant circuits was 250 mW with a coil displacement of 0.7 to 1.5 cm. Si *et al.* [53] designed a wireless power supply system for implantable biomedical devices. The design reduced the losses and showed tolerance against load variation.

Chaimanonart *et al.* [54] developed a wireless implantable system implanted inside the skin of small laboratory mice. The power source adjusted its RF power, strength and achieves a stable and reliable voltage supply for the bio-implant system. Hyung *et al.* [55] presented a Class E PA with an automatic power control loop and load compensation circuit. The system provided an output power of 10–30.2 dBm with efficiency of 71.5%. Silay *et al.* [56] made a closed-loop control for power transfer circuit by controlling the supply voltage. The efficiency of the inductive link was 55%.

In 2011, Andia *et al.* [57] used a closed loop to control voltage supplied to a PA by using a DC/DC converter. At 20 mW implant power consumption the measured efficiency was 69%. Pablo *et al.* [58] controlled and regulated the DC voltage in an implanted device. The efficiency of the system increases using amplitude voltage control for mutual displacement between coils of 0–11.8 mm. Frequency control was improved too. Hongcheng *et al.* [59] improved the transcutaneous closed loop power management efficiency. The controller achieved the best transfer in case of the coupling variation; the system achieves high efficiency even with loss in the compact components such as the PCB pattern coil and the CMOS switch [60]. The output voltage is kept constant under distance and load variations. Summaries of frequency, power consumption, coils displacement and efficiency for power control are shown in Table 3.

#### 3.1.3. Voltage Controlled Oscillator (VCO) with Closed Loop Technique

**Figure 14.**
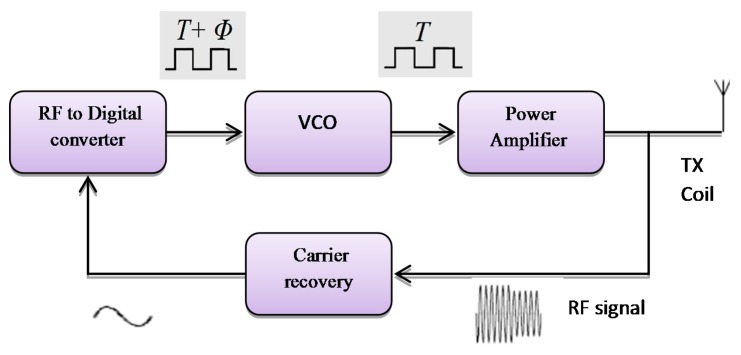
Voltage controlled oscillator.

A different method which can be implemented for monitoring the RF transmitter, uses feedback control techniques. That follows a design of a simple control system to auto-correct the RF signal using VCO frequency that offers output power stability for the power amplifier as shown in Figure 14. It provides constant voltage at the implant device [61]. This method is simple to implement to reduce the reader hardware. The feedback system consists of an amplifier for the RF signal passed through a square circuit (multiplier) filtered at the center frequency. The RF signal is converted into the digital signal, which converts the captured transmitted signals in the digital form. The phase comparator is used to compare the digital transmitted signal with respect to the fundamental transmission pulse signal by VCO.

The voltage controlled oscillator (VCO) was used by Gyu and Cho in 1998 [62]; they designed a transcutaneous inductive coupled system that power an artificial heart. Design reduced the effects of variations in coupling coefficient and changing with load. Shabou *et al.* [63] used numeric control operating with a VCO (voltage controller output). Results showed approximately zero power loss while switching. Mizannojehdehi *et al.* [64] used a transcutaneous power system with frequency controller. Results showed that self-tuning ability while the system is operating led to high efficiency and more reliability. Walia and Drakakis [65] designed a 13.56 MHz frequency shift keying (FSK) short-range (7–8 cm) biotelemetry link. Design of a PLL with a VCO control loop at 13.56 MHz was applied.

In 2011, Betancourt *et al.* [66] built a low power RF transmitter suitable for near field implantable devices. Aqueveque *et al.* [67] developed a frequency controller by using a VCO to control the operating frequency of the system. Regulated voltage was stable when the distance between coils varies between 0 mm and 40 mm. Results show that it is not necessary to increase the supply voltage of the external unit to regulate the internal voltage. Adeeb *et al.* [68] designed a biotelemetry system, demonstrating power transmission of 125 mW with 12.5% power link transmission efficiency. Summaries of frequency, power consumption, coils displacement and efficiency for the VCO frequency controller are shown in Table 4.

#### 3.1.4. Capacitor Control Operating Frequency Method

**Figure 15.**
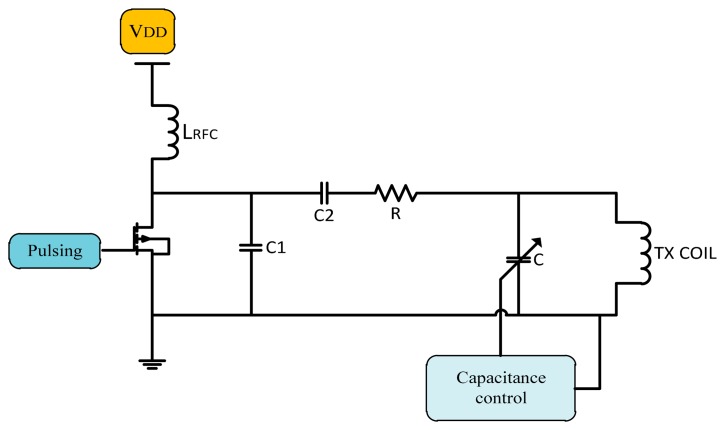
A capacitor used to control resonance frequency.

There are other methods to stabilize the system operating frequency using a variable capacitor fixed in parallel with transmitter coil whose equivalent capacitance is controlled by semiconductor switches as shown in Figure 15.

In 2005, a variable capacitor used to control the resonance frequency by Si *et al.* [69] to regulate the transcutaneous power by adjusting the resonant frequency of the primary elements. Results monitored a power efficiency of 80% at 1 cm gap demonstrating that the proposed method is very effective in stabilizing the operating frequency, while maintaining the zero voltage switching operation. Ping Si *et al.* [70] used a method for adjusting the resonant operating frequency of the primary part. Operating frequency was changed by effective tuning capacitance, and by soft switching the heating at the secondary coil was reduced. The result shows the need for better thermal management on the primary side.

Dissanayake *et al.* 2009 [71] used closed loop frequency control system with a switched capacitor control method and power regulated for separations of 10 mm to 20 mm at 10 W. Mohamadi [72] changed the operating frequency by controlling the tuned capacitor on the primary side, according to the feedback signals. Shmilovitz *et al.* [73] used a method where voltage regulation is based on hysteretic control of the implanted storage capacitor voltage. The results show the improvement in system response to the change of load. Summaries of frequency, power consumption, coils displacement and efficiency for capacitance frequency control are shown in Table 5.

#### 3.1.5. Microcontroller Operating Frequency

**Figure 16.**
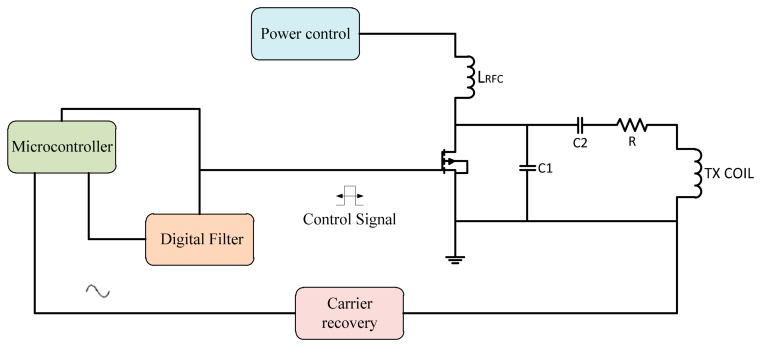
A microcontroller used to control frequency shift.

Another method is using microcontrollers as more flexible tools, whether the programming code use assembler or C languages. A modulation technique for controlling the fundamental carrier signal led to an increase of the system performance efficiency. Many techniques for generating the control signal use microcontrollers so the application of this facility in microcontrollers for generating the control signal offers flexibility to update the data and auto-correction for the ASK signal or PWM signal [74]. By introducing the feedback control for the power amplifier transmitted signal, modifying the design for the controller to control the fundamental carrier signal that increased the stability for Class E power amplifiers. Figure 16 shows a frequency control system using a microcontroller.

The automatic controller circuit was introduced for improving the transmitter power to the implant device. That offer a stability of transfer of the wireless power and provides minimum shifting of the carrier frequency. The microcontroller is generating a control pulse signal, this transmitted signal captured by the loop coil circuit is amplified and then compared to the filtered transmitted carrier signal. The microcontroller converts them into digital form for the compactor, and the error signal is fed back into the control signal generator to correct the output.

The microcontroller technique used by Kiani and Ghovanloo [75] developed a closed-loop transcutaneous system operating at 13.56 MHz. The coils' distance was changed between 5–20 mm. using microelectronic devices (IMDs) to control the system. Kuanfu *et al.* [76] designed an integrated 256 channel IC. Integrated digital controllers control the power and operating frequency of the system. Tsujimura *et al.* [77] designed a microcontroller, while the external unit also has a converter. The system could monitor and control the conditions of a patient's artificial heart. Paglinawan *et al.* [78] used a microcontroller unit (MCU) that was configured to control the whole system.

Lee *et al.* [79] in 2011 designed a programmable implanted stimulator, so the power control unit consists of a rectifier, battery charging and detection and a regulator. A PLL-based phase shift keying demodulator was used to stimulate data and adjust the clock. Choi *et al.* [80] used a resonant regulated rectifier with carrier frequency of 6.78 MHz that delivers 6 W power and efficiency of 86%. Li *et al.* [81] designed a 13.56 MHz wireless power transfer system with a 1X/2X reconfigurable resonant regulating (R3) rectifier. A PWM loop on the secondary side controls the duty cycle of switching the rectifier and configures load and coupling variations. Summaries of frequency, power consumption, coils displacement and efficiency for microcontroller frequency control are shown in Table 6.

## 4. Issues and Challenges

Recently, it has become necessary for wireless communication systems to be used in biomedical implanted devices. Therefore, circuits with compact design, lowest cost, and low power loss transfer occupy most of the attention of researchers. In biomedical implanted devices the delivered power and the stability of DC supplied voltage to the implanted device are very important to make the device work in an optimum state while ensuring patient safety. The misalignment between transfer coils (external and internal) in any direction *X*-axis or *Y*-axis or angular misalignment or due to load variation results in a frequency shift and power losses. To achieve a low-cost and highly reliable transcutaneous power system, there are parameters that must be achieved and investigated like using a sufficiently high efficiency power amplifier with suitable transfer coils, good modulation, using biomedical frequency bands and good control technique.

### 4.1. Efficient Transcutaneous Power Amplifiers

To achieve low-cost and highly reliable transcutaneous wireless transmission with high power and high efficiency the use of power amplifiers (PAs) is necessary. High efficiency PAs are commonly used switch-mode topologies, such as Class D, E, and F. The CMOS process offers the highest integration level at the lowest cost and with smaller size. CMOS transistors possess enough speed to implement RF circuits and they offer the highest density digital circuits. Power efficiency is an important factor in the design of the power amplifier (PA) [2]. The power efficiency of switched mode PAs like Class D, E and F, amplifiers have a better transfer efficiency compared with these linear amplifiers, such as Class A, AB, B, and C, especially at high frequencies, wide bandwidth and low power losses.

### 4.2. Coil Size

Power supply and antenna space limitations are important in implant antenna design. Many factors are considered in antenna design, such as low signal power levels, reduced space availability and the effect that the tissue characteristics have on the antenna. Both transfer coils should be adjusted to the same resonance frequency so maximum power transfer occurs [27]. Efficiency of coils can be increased by antenna design techniques and by modifying their lumped components to cancel reluctance and match dissimilar impedances, however, these elements increase antenna loss. The use of low frequencies in small antennas is also impractical, that it has greater signal attenuation in body tissues, therefore a specific biomedical frequency band required, taking into consideration the lack of increase of tissue heating [82]. The main reason for the power dissipation is the implanted coil. Therefore, the coil shape and geometry should be effective and as small as possible with the lowest misalignments and lowest power dissipation possible [83]. To overcome the above issue, the design and optimization of efficient inductive power links with small size have been well studied in [84,85].

### 4.3. Suitable Carrier Frequency

The carrier frequency is very important in designing implanted devices and biotelemetry systems. Most of the implantable devices are powered by a low frequency, less than 1 MHz, however, the standard safety level with respect to human body exposure to radio frequency electromagnetic fields is 3 kHz–30 GHz [86].

The main drawback of inductive links is that the low frequency limits the available bandwidth which results in low data rates. To overcome range limitations, a dedicated frequency band has been allocated for medical implantable device communication system (MICS), which uses the frequency band of 402–405 MHz, which involves a number of allowable frequencies such as 27 MHz and 216–217 MHz. The MICS solves the range limitations associated with inductive links. They also have good signal propagation characteristics in the human body. Also, the standard frequencies of the Industrial, Scientific and Medical (ISM) band were applied like 125 KHz, 135 KHz, 6.678 MHz, 13.56 MHz, 20 MHz, 27 MHz and 47 MHz [87]. The ISM has different frequency bands, with the most used being the 2.4 GHz and sub-1 GHz band. The 2.4 GHz band is known for its high speed. These frequencies are used to protect the human from hurt by spoiling the skin tissue or any side effects.

The low and high frequencies between 3 kHz–30 MHz are widely used in transcutaneous wireless due to its ability penetrate water and skin over a short range and the fact they cannot heat the surrounding biological tissue, which is tested by *Mutashar et al.* [82].

### 4.4. Modulation Technique Consideration

One biomedical system operation is the transmission of sensor data in real time for monitoring and diagnosis. Many biotelemetry modulations such as amplitude shift keying (ASK), frequency shift keying (FSK) and pulse shift keying (PSK) are used. Important parameters needed for the transcutaneous power transfer system are wide bandwidth, compact design, low power losses, secured data, and best signal quality. Those parameters ensure good performance over the transmission channel and reduce noise and distortion. The ASK modulation is considered as the simplest modulation used in near-field transcutaneous power systems. However, for more immunity against interference, noise reduction and high data rate (PSK) is used [88].

There are many methods to conserve power and reduce the interference on nearby electronics by choosing a suitable operated frequency according to the ISM band, suitable modulation and suitable design of the modulator and demodulator. The detailed literature review of this chapter is based on modulation forms technique which is used in the implanted devices. These modulation techniques are amplitude shift keying (ASK), frequency shift keying (FSK) and phase shift keying (PSK) [89]. In general the ASK modulation technique is widely used in implanted devices due to its simplicity and low power consumption. However, it has a number of limitations for high-bandwidth data transmission; and high order filters with a sharp cut off frequency [1,90].

### 4.5. Frequency Control Techniques

The goal is to monitor and control the transmitter carrier frequency of the power amplifier by using a frequency control technique which offers a high transfer wireless power stability that maintains the stability of DC voltages at the implant device. The technique used should satisfy the general requirement of transcutaneous systems which are high power transfer efficiency, high data rate, compact design, low cost and safety. Each of one of the techniques mentioned in this paper has advantages and disadvantage according to these requirements. The use negative feedback with a control technique, allows the transmission system to have a constant transfer voltage, adjustable duty ratio and no frequency shift in the transmitted signal. The effectiveness of the negative feedback technique was tested in many control techniques with a variable load and variable displacement between coils. The experimental results show that the power amplifier with feedback loop can keep operating in a better state under these conditions [44].

### 4.6. Transmitter Chip Size

The use of an automatic control circuit is introduced to improve the power transmitted toward the implant device. That offers stability and minimum shifting for the carrier frequency. This controller and related feedback circuitry increase the chip size of the external part of transcutaneous transmission system, so the power dissipation generated by the circuit increases as heat. The lower system cost and small form factor, the digital, analog and RF circuitry should be integrated onto a single die. Recently technologies like the CMOS process are used to fabricate chips. CMOS transistors have the speed to implement RF circuits and have high density digital circuits. Fabrication using standard 0.35 μm CMOS technology was widely used for its high speed, low cost, low power consumption.

## 5. Conclusions and Suggestions

This work explains the details of the various biomedical power amplifier operating frequency control techniques. Wireless inductive coupling technology for biomedical applications was used which transmits a low power, which is less than a milliwatt. Radiated, radio frequency (RF) power signals from reader coil antennas deliver a stable wireless power transmission. The best RF signal quality makes DC voltage at the implant device stable, independent of distances between coils. The feedback technique used to develop the transmitted signal from the external part coil of the biomedical implanted device to the receiver coil implanted inside the body (internal part). Maximum power transfer efficiency of inductive coupling link is done by adjusting both sides of the cooling coils at the same resonant frequency *f*_0_.

The misalignment or change in distance between the coils will cause a carrier frequency shift. Class E power amplifiers are commonly used for transcutaneous power transmission because of their simple circuit design, high efficiency, and reliability. The operating point of the Class E amplifier will be shifted and cannot adjust frequency, duty cycle and there will be power dissipation in the switching device and device distortion. The feedback frequency link will reduce power consumption, keep the resonant frequency stable and control frequency shifts. That feedback signal is used to control transistor gate switching of the power amplifier or power control. This study focused on the existing frequency feedback control methods used in biomedical implant devices and their results, disadvantages and present challenges to satisfy the needs for high efficiency, low cost, simple circuit design and high data transmitted rate. To achieve the abovementioned a kind of artificial intelligence based on an automatic frequency controller is used to deliver stable signals with low losses.

## Figures and Tables

**Table 1.** Power amplifier's efficiency, advantage and disadvantage.

**Power Amplifier Type**	**Efficiency Theoretical**	**Applications**	**Advantage**	**Disadvantage**	**References Efficiency**
A	50%	RF applications	-	-	[31]
B	78.5%	RF applications	-	-	[31]
AB	70%–80%	RF applications	-	-	[31]
C	83%–90%	RF applications	-	-	[31]
D	80%	RF and bio-telemetry applications	Has a better response to the inductively coupled even in the case of frequency shifting	Requires two active devices, limited operating frequency and provide low power transmission efficiency	[22]
E	90%–95%	RF and bio-telemetry applications	Simple architecture and need only one active device, the frequency range is wide KHz-MHz and have high power transmission efficiency	Any shift in resonance frequency will decrease the output power transmission appreciably	[2,28]
F	90%–100%	RF and bio-telemetry applications	Need only one active device, the frequency range is wide KHz-MHz and have higher power transmission efficiency	Any shift in resonance frequency will decrease the output power transmission appreciably and need one more inductor	[18]

**Table 2.** Gate drive switching control method used in transcutaneous amplifier control.

**Technique**	**Carrier (MHz)**	**CMOS (μm)**	**Power Consumption**	**Efficiency**	**Power Supply (V)**	**Displacement (mm)**	**Year [Reference]**
Gate control	0.760	-	-	-	9	-	1992 [36]
Gate control	20	0.35	-	-	10	-	1999 [37]
Gate control	4.5	-	1–10 mW	74%–54%	3	1–10	2007 [38]
Gate control	0.25–0.32	-	-	-	30–60	10–20	2009 [39]
Gate control	1	-	-	44.3%–83.4%	6	-	2009 [40]
Gate control	0.155–0.168	-	-	82.1%	-	16	2009 [41]
Gate control	1	-	-	-	6	-	2010 [42]
Gate control	0.3–0.4	-	-	-	30–45	10–20	2010 [43]
Gate control	1	-	-	67.6%	6	40–69	2010 [44]
Gate control	0.120	0.8	-	-	5	-	2012 [45]
Gate control	13.56	0.5	-	77%	-	-	2012 [46]
Gate control	0.083–0.175	-	-	66.2%–30%	5	2–8	2012 [47]
Gate control	8	130 nm	-	-	4–6	-	2013 [48]
Gate control	0.160	-	-	93.4%	-	0–15	2013 [49]
Gate control	14	0.18	-	80%	5–30	-	2014 [50]

**Table 3.** Power supply control method used in transcutaneous amplifier control.

**Technique**	**Carrier (MHz)**	**CMOS (μm)**	**Power Consumption**	**Efficiency**	**Power Supply (V)**	**Displacement (mm)**	**Year [Reference]**
Power control	1	0.16	-	-	15	4–15	2004 [51]
Power control	1	1.5	-	65.8%–36.3%	-	7–15	2005 [52]
Power control	0.155	-	-	-	10	-	2007 [53]
Power control	4	0.15	-	-	2	-	2008 [54]
Power control	-	0.35	-	71.5%	5	-	2011 [55]
Power control	8	0.18	-	-	3.3	10	2011 [56]
Power control	13.56	-	20 mW	69%	-	-	2011 [57]
Power control	1	-	-	-	5	0–11.8	2012 [58]
Power control	13.56	0.35	-	80%	-	-	2012 [59]
Power control	8	0.35	-	63%	3	10–20	2014 [60]

**Table 4.** VCO method used in transcutaneous amplifier control.

**Technique**	**Carrier (MHz)**	**CMOS (μm)**	**Power Consumption**	**Efficiency**	**Power Supply (V)**	**Displacement (mm)**	**Year [Reference]**
VCO	0.1225–0.1733	-	-	-	-	10–20	1998 [62]
VCO	10.8–13.2	0.35	-	-	3.3–4.5	-	2005 [63]
VCO	0.385–0.408	-	-	73%	14	10–24	2006 [64]
VCO	13.56	0.8	-	-	-	70–80	2007 [65]
VCO	174–216	0.5	-	-	-	-	2011 [66]
VCO	0.950–1.2	-	-	-	2.3–5.5	6–20	2012 [67]
VCO	0.2	-	-	-	-	-	2012 [68]

**Table 5.** Capacitance control method used in transcutaneous amplifier control.

**Technique**	**Carrier (MHz)**	**CMOS (μm)**	**Power Consumption**	**Efficiency**	**Power Supply (V)**	**Displacement (mm)**	**Year [Reference]**
Capacitance control	0.087	-	-	-	10	-	2005 [69]
Capacitance control	0.078	-	-	80%	10	10	2008 [70]
Capacitance control	0.163–0.173	-	-	-	23.5	10–20	2009 [71]
Capacitance control	0.1	-	-	-	6	-	2011 [72]
Capacitance control	0.75	-	-	-	-	20–85	2014 [73]

**Table 6.** Microcontroller used in transcutaneous amplifier control.

**Technique**	**Carrier (MHz)**	**CMOS (μm)**	**Power Consumption**	**Efficiency**	**Power Supply (V)**	**Displacement (mm)**	**Year [Reference]**
microcontroller	13.56	-	78 mW–1.1 W	-	3.6	5–20	2010 [75]
microcontroller	8	0.18	-	-	36	-	2010 [76]
microcontroller	2.4 GHz	-	-	-	-	-	2010 [77]
microcontroller	10	0.35	0.39 mW–0.745 mW	-	3–4.5–15	-	2011 [78]
microcontroller	4	0.35	-	-	-	-	2011 [79]
microcontroller	6.78	0.35	-	86%	10–14	-	2013 [80]
microcontroller	13.56	0.35	-	92.6%	-	-	2014 [81]
